# High-throughput sequencing-based analysis of the composition and diversity of the endophyte community in roots of *Stellera chamaejasme*

**DOI:** 10.1038/s41598-024-59055-x

**Published:** 2024-04-13

**Authors:** Jun-ze Zhang, Xin-zhong Li, Ye-bing Yin, Si-cen Luo, Dong-xu Wang, Heng Zheng, Yi-xuan Liu

**Affiliations:** 1 Key Laboratory of Biodiversity and Environment on the Qinghai-Tibetan Plateau, Ministry of Education, School of Ecology and Environment, Tibet University, Lhasa, China; 2 School of Resource and Environmental Engineering, Wuhan University of Technology, Wuhan, China

**Keywords:** *S. chamaejasme*, Endophytic bacteria, Endophytic fungi, Community structure, Composition differences, Microbial ecology, Environmental microbiology

## Abstract

*Stellera chamaejasme* (*S. chamaejasme*) is an important medicinal plant with heat-clearing, detoxifying, swelling and anti-inflammatory effects. At the same time, it is also one of the iconic plants of natural grassland degradation in northwest China, playing a key role in the invasion process. Plant endophytes live in healthy plant tissues and can synthesize substances needed for plant growth, induce disease resistance in host plants, and enhance plant resistance to environmental stress. Therefore, studying the root endophytes of *S. chamaejasme* is of great significance for mining beneficial microbial resources and biological prevention and control of *S. chamaejasme*. This study used Illumina MiSeq high-throughput sequencing technology to analyze the composition and diversity of endophytes in the roots of *S. chamaejasme* in different alpine grasslands (BGC, NMC and XGYZ) in Tibet. Research results show that the main phylum of endophytic fungi in the roots of *S. chamaejasme* in different regions is Ascomycota, and the main phyla of endophytic bacteria are Actinobacteria, Proteobacteria and Firmicutes (Bacteroidota). Overall, the endophyte diversity of the NMC samples was significantly higher than that of the other two sample sites. Principal coordinate analysis (PCoA) and permutational multivariate analysis of variance (PERMANOVA) results showed significant differences in the composition of endophytic bacterial and fungal communities among BGC, NMC and XGYZ samples. Co-occurrence network analysis of endophytes showed that there were positive correlations between fungi and some negative correlations between bacteria, and the co-occurrence network of bacteria was more complex than that of fungi. In short, this study provides a vital reference for further exploring and utilizing the endophyte resources of *S. chamaejasme* and an in-depth understanding of the ecological functions of *S. chamaejasme* endophytes.

## Introduction

Plant endophytes mainly refer to fungi and bacteria that are symbiotic or parasitic in healthy plants^1^. They do not cause apparent lesions in plant tissues^2,3^, can synthesize substances needed for plant growth^4^, induce disease resistance in host plants^5^, and enhance plant resistance to environmental stress^6^. Some endophytes can be isolated from plant tissues undergoing rigorous surface disinfection. Many studies have shown that the composition of plant endophytes is usually closely related to the plant growth environment^7,8^. Current research on the plant microbiome mainly focuses on the interaction of rhizosphere microbial communities with plants to adapt to the surrounding environment^9^. Compared with rhizosphere microorganisms, endophytes may have a closer relationship with plants in terms of environmental adaptation. In addition, endophytes are also involved in the growth and development of plants and have a particular impact on the specific responses of plants^10^. They can affect the growth of plants by producing various hormones or acting as transporters of minerals^11,12^, and they can also secrete specific compounds to inhibit toxic substances in plants^13,14^. Studies have shown that under different salinity, pH, light, photoperiod and temperature conditions, endophytic fungi can significantly improve the germination rate and survival rate of *Oxytropis glabra*seeds and, at the same time, enhance resistance to environmental stress^15^. A large number of studies have shown that plant endophytes play a crucial role in the growth and development of plants and are involved in plant growth under specific conditions^16^. Endophytes can provide nitrogen to plants through nitrogen fixation metabolism^17^, produce phytohormones to regulate plant growth and development^18^, and some endophytes can even affect the plant's gene expression to enhance adaptation to the external environment^19^. For example, several strains of endophytic bacteria isolated from *Oxytropis glabra* not only have sound antibacterial effects against Fusarium wilt of potato but can also fix nitrogen and produce indoleacetic acid^20^. Yang et al.’s research on endophytic fungi of *Stipa purpurea* on the Qinghai-Tibet Plateau showed that *Stipa purpurea* possesses endophytic fungal resources rich in potential for active secondary metabolites^21^. On the other hand, in the study of endophytic bacteria in *Stipa purpurea*, it was found that the endophytic bacterium *Bacillus subtilis* can help the plant maintain the stability of biochemical indicators under stress and improve its ability to resist stress^22^. In addition, Plants can also recruit microorganisms from the surrounding soil environment by regulating the secretion of metabolites, and these microorganisms subsequently enter the plant through the root system to help the host resist stress from environmental changes^23^. Furthermore, some endophyte-derived secondary metabolites may be important sources for the discovery of drugs to treat various diseases^24^. Therefore, the study of endophytes in the roots of medicinal plants has great significance for the in-depth exploration of the interactions between plants and external environmental factors, the physiological mechanisms of plants, the interactions between medicinal plants and external environmental factors, and the synthesis of metabolites.

*S. chamaejasme* is a perennial herbaceous plant whose whole plant is toxic^25^ and is widely distributed in northwest China. In addition, *S. chamaejasme* is a traditional Chinese medicine that has the effects of clearing away heat and detoxifying, reducing swelling, and healing ulcers^26^. However, in recent years, the expansion of *S. chamaejasme* in degraded grasslands in northern China has led to the gradual replacement of original dominant plants, seriously accelerating the degradation of natural grasslands in northern China and limiting the development of animal husbandry. Therefore, it has become one of the iconic plants of grassland degradation in northern China. At present, research on *S. chamaejasme* mainly focuses on morphology^27^, ecology^28^, chemicals^29^ and biological control^30^. When *S. chamaejasme* is used for medicinal purposes, the main part used is its root, and a large number of studies on its medicinal ingredients have mainly focused on the root^31,32^. At the same time, roots are also important organs of plants, not only able to absorb nutrients from the soil, but also play a vital role in improving the survival of plants under various external environmental conditions^33^. However, there are relatively few studies on the endophyte composition of *S. chamaejasme*, especially endophytic bacteria^30^. Jin et al. found that Sphingomonas bacteria that can produce active substances such as gibberellin and auxin exist in the rhizosphere soil and roots of Diphragma rubra at different altitudes on the Qinghai-Tibet Plateau^34^. With the rapid development of biotechnology, traditional microbial isolation and identification methods can no longer meet the needs of large-scale and diverse microbial groups. As a second-generation sequencing technology, high-throughput sequencing technology can directly sequence large-scale gene sequences, so it has been widely used in studying plant endophyte diversity^35^. Jin et al. also analyzed the root endophytic fungi and bacterial communities of *S. chamaejasme* in Cuiying Mountain and Min County, Gansu Province, by constructing a clone library. The results showed that root endophytic fungi mainly belong to the phyla Ascomycota and Basidiomycota, while the bacteria mainly belong to Proteobacteria and Actinobacteria^36,37^. However, current research on endophytes associated with *S. chamaejasme* roots is limited. Except for sporadic reports on endophytes and rhizosphere microorganisms in Gansu Province, China^38,39^, there have been no reports on endophytes in alpine grasslands in Tibet. Therefore, studying the endophytes of *S. chamaejasme* in different alpine grasslands of Tibet is of great significance for understanding the root endophyte resources of *S. chamaejasme* and its adaptation to the plateau environment.

In this study, we used high-throughput sequencing technology to study the composition and diversity of endophytic fungal and bacterial communities in the roots of *S. chamaejasme* at three locations in the Tibet, China. We analyzed the changing patterns of root endophytes of *S. chamaejasme* in different regions to reveal the characteristics of the root endophyte community of *S. chamaejasme*, a toxic weed in alpine grasslands, and provide a theoretical basis for the ecological restoration of the Qinghai-Tibet Plateau and the development of beneficial microbial resources.

## Materials and methods

### Sample collection and treatment

In total, 30 samples of *S. chamaejasme* were collected in 2023 from three locations: BGC in Bange city, NMC in Dangxiong city, and XGYZ in Shenzha city (Table 1). Professor Yi-xuan Liu of Tibet University identified *S. chamaejasme* based on the "Flora of China", and the specimens are stored in the herbarium of the School of Ecology and Environment of Tibet University. During sampling, 30 healthy roots of *S. chamaejasme* were selected and collected from sample sites. Subsequently, the samples were loaded into sterile sampling bags, marked, placed in a car refrigerator at 4 °C, and processed within 24 h. The samples’ latitude and longitude coordinates were used in the World Geodetic System, 1984 (WGS-84) and were recorded using a hand-held GPS unit (Etrex 221x, Garmin, CH.). Samples of roots of *S. chamaejasme* were rinsed with tap water and placed on an ultraclean table for surface sterilization. The specific method was as follows: soaked in 75% alcohol for 3 min, rinsed with prepared sterile water 3–5 times, soaked in 5% NaClO for 3 min, rinsed with prepared sterile water 3–5 times, soaked in 75% alcohol for 2 min, and rinsed with prepared sterile water 3–5 times. The last prepared sterile water was used to coat the plate, and the surface disinfection effect was tested. The surface sterilized roots of *S. chamaejasme* were cut into small pieces, put into sterile 2 mL centrifuge tubes and stored at − 80 °C until use.

**Table 1:** Sampling information of *S. chamaejasme.*

Sample id	Longitude (°E)	Latitude (°N)	GPS coordinates	Date
BGC	89.40702166	31.78202451	WGS-84	2022.08.07
NMC	90.99014848	30.92840938	WGS-84	2022.08.10
XGYZ	88.7198288	31.68645035	WGS-84	2022.08.05

### DNA extraction and high-throughput sequencing

The sample DNA was extracted from the filter membranes using a Power DNA Isolation Kit (Qiagen, Germantown, MD, USA) according to the manufacturer’s protocol. DNA quality was checked using 1% agarose gel electrophoresis. DNA concentration and purity were determined with a NanoDrop 2000 spectrophotometer (Thermo Fisher Scientific, Wilmington, DE, USA). Next, the ITS1F-ITS2R region of the fungus was amplified by PCR with the primers ITS1F (CTTGGTCATTTAGAGGAAGTAA) and ITS2R (GCTGCGTTCTTCATCGATGC). The bacterial 16S rDNA V3-V4 hypervariable region was PCR-amplified using the following primers: 799F (AACMGGATTAGATACCCKG) and 1392R (ACGGGCGGTGTGTRC). The fungal PCR reaction conditions were: 5 min at 95 °C; 20 cycles of 95 °C for 45 s, 57 °C for 30 s, and 72 °C for 30 s; and 72 °C for 10 min. Bacterial PCR reaction conditions were: 95 °C for 5 min; 20 cycles of 95 °C for 45 s, 57 °C for 30 s, and 72 °C for 30 s; and 72 °C for 10 min. The amplified products were purified and mixed in equivalent amounts. PCR products were sequenced using the PE250 strategy on the Illumina MiSeq 2500 platform by Majorbio (Shanghai, China).

### Bioinformatics and statistical analysis

After quality filtering the raw data, high-quality clean data were received for subsequent analysis. The clean data were demultiplexed separately by their unique barcodes. A standard denoising pipeline was used to obtain the amplicon sequence variants (ASVs) using the DADA2 plug-in in QIIME2 software (version 2022.8)^40^, after which an ASV abundance table was constructed. The ASVs were annotated using the SILVA database (version 138)^41^. Low-abundance ASVs (< 10 reads) were removed. Three replicates were used to reduce the sampling bias. The ASV table was then rarefied to 40,000 reads per sample for downstream analysis. Alpha diversity indices of endophytic bacterial communities, including the richness index, Shannon‒Wiener diversity index, Chao1 index, ACE index and Simpson dominance index, were calculated using the “vegan” packages in R software (version 4.1.1). Principal coordinate analysis (PCoA) and PERMANOVA were performed based on the Bray–Curtis distance using the “vegan”, “micro eco” and “ggplot2” packages in R software. Co-occurrence patterns of endophytic bacteria and fungal communities were constructed based on Spearman's rank correlation coefficients. Co-occurrence events were identified as statistically robust correlations (*|R|*> 0.6, *P* < 0.05), and the co-occurrence network was visualized in Gephi (version 0.10.1).

### Ethics approval and consent to participate

Licensed by the School of Ecology and Environment of Tibet University, we comply with all relevant institutional, national and international guidelines. No materials from animals or humans were used in this study. Our experimental studies, including collection of plant material, comply with institutional, national or international guidelines. The collection of *S. chamaejasme* samples was permitted by the Forestry and Grassland Bureau of the Tibet Autonomous Region. Research conducted on plants complies with relevant regulations and guidelines.

## Results and discussion

### High-throughput sequencing statistics and endophytic community diversity

**Figure 1:**
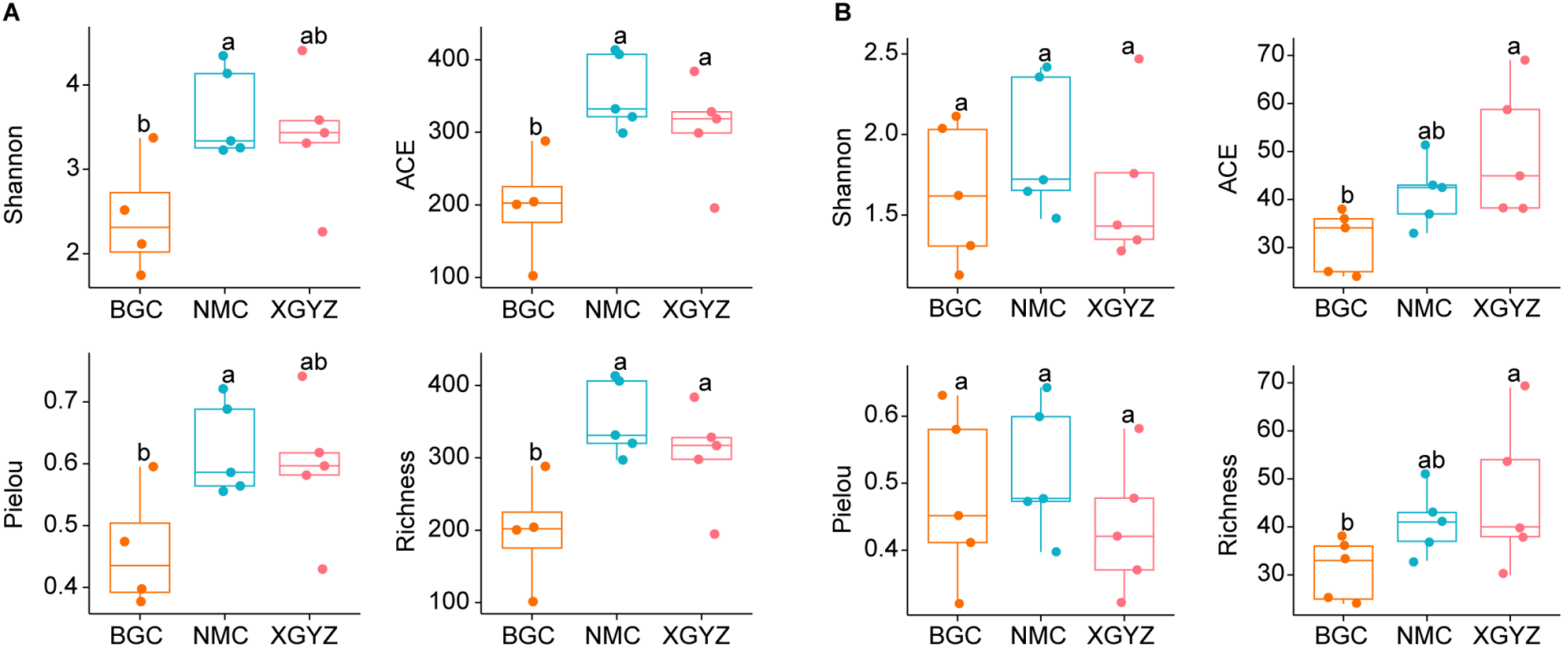
Comparison of Alpha Diversities Among Different Regions. (**A**) Bacterial Community. (**B**) Fungal Community. The boxplot displays the range from the first quartile to the third quartile. The median is represented by the black line, while filled circles denote individual sample values. Different letters indicate significant differences based on ANOVA (*p* < 0.05).

A total of 30 samples were collected, with 10 samples each from the BGC, NMC, and XGYZ regions. Amplicon sequencing technology was used to sequence the V3-V4 region of the bacterial 16S rRNA gene and the fungal ITS rRNA gene. For the bacterial community, we initially detected a total of 991,111 reads. After rigorous filtering to remove low-quality and chimeric sequences, we retained 876,297 high-quality sequences. These sequences were subsequently classified into 1,868 amplicon sequence variants (ASVs) using the DADA2 pipeline. For the fungal community, 1,250,650 reads were assembled, resulting in 9,120,801 high-quality sequences. These sequences were further classified into 323 ASVs. The rarefaction curves approached the saturation plateau, suggesting that the sequencing depths were sufficient to cover bacterial and fungal diversity (Supplementary data Fig. S1). For the bacterial community, NMC exhibited the highest Shannon diversity index, ranging from 3.22 to 4.34, followed by XGYZ with a range of 2.27 to 4.42 and BGC with a range of 1.74 to 3.37. Similarly, the ACE index ranged from 299.01 to 413.52 in NMC, 195.79 to 384.00 in XGYZ, and 102.34 to 288.00 in BGC. The Pielou index showed similar trends, ranging from 0.55 to 0.72 in NMC, 0.43 to 0.74 in XGYZ, and 0.38 to 0.60 in BGC. The richness index also exhibited consistent patterns, ranging from 297 to 413 in NMC, 195 to 384 in XGYZ, and 101 to 288 in BGC (Fig. 1A and Table S1). Both the Shannon and Pielou indices exhibited similar patterns for the fungal community. The Shannon index ranged from 1.48 to 2.42 in NMC, 1.12 to 2.12 in BGC, and 1.28 to 2.46 in XGYZ. Similarly, the Pielou index ranged from 0.40 to 0.64 in NMC, 0.32 to 0.63 in BGC, and 0.32 to 0.58 in XGYZ. The Richness and ACE indices also displayed consistent trends. The richness index ranged from 30 to 69 in XGYZ, 33 to 51 in NMC, and 24 to 38 in BGC, while the ACE index ranged from 38.17 to 69.00 in XGYZ, 33.00 to 51.35 in NMC, and 24.00 to 38.00 in BGC (Fig. 1B and Table S2).

### Composition of the endophyte communities in *S. chamaejasme* roots

**Figure 2:**
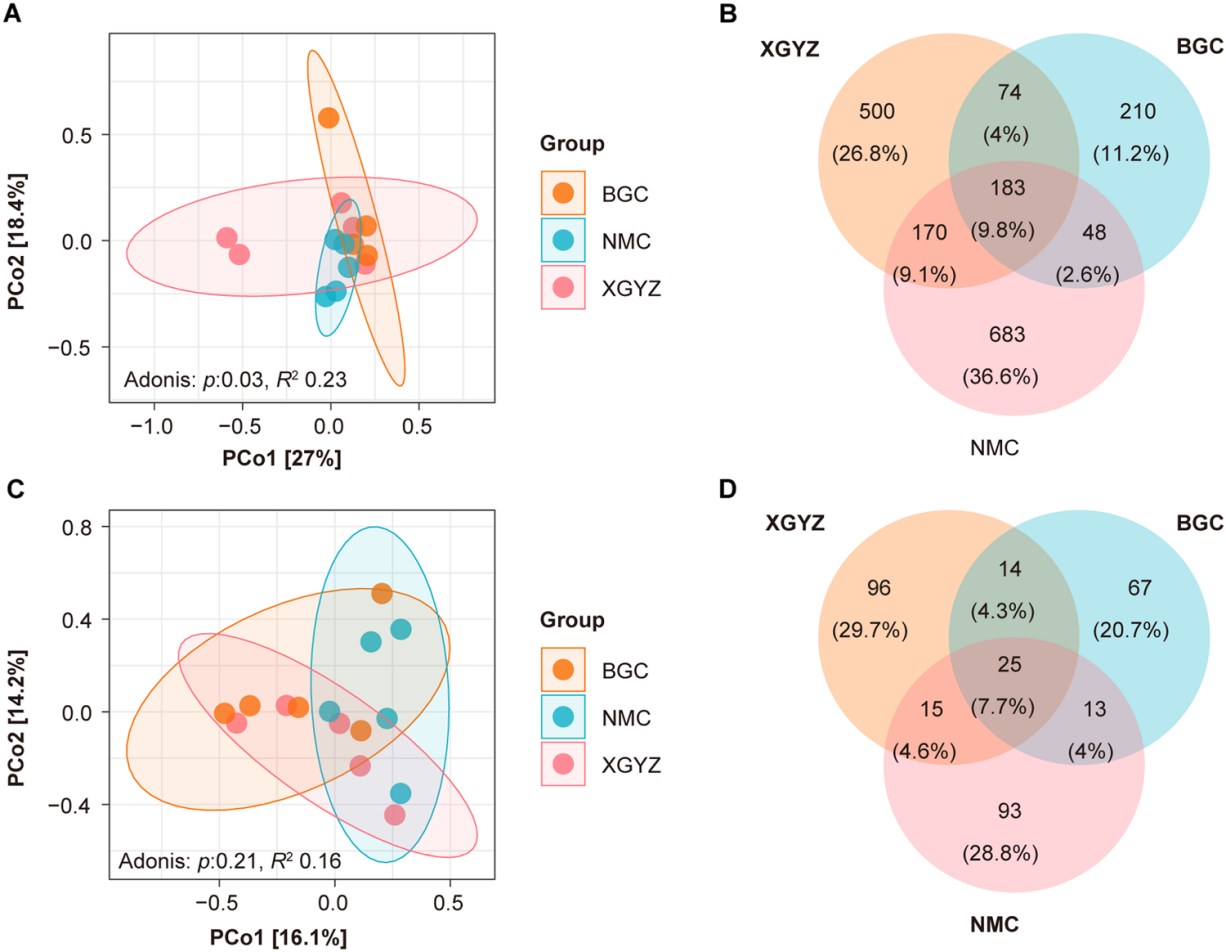
Composition of endophytes in *S. chamaejasme* root samples. (**A**) Multiple sample principal coordinate analysis (PCoA) of the ASV level in the bacterial community. (**B**) Venn diagram showing the number of shared and specific ASVs in the bacterial community for each region. (**C**) Multiple sample principal coordinate analysis of the ASV level in the fungal community. (**D**) Venn diagram showing the number of shared and specific ASVs in fungal communities for each region.

We analyzed the composition of the bacterial and fungal communities in the BGC, NMC, and XGYZ regions. Principal coordinate analysis (PCoA) and PERMANOVA revealed significant differences in the composition of both bacterial and fungal communities among the BGC, NMC, and XGYZ samples. The results showed that PCoA1 and PCoA2 explained 27.0% and 18.4% of the total variation in bacterial communities and 16.1% and 14.2% in fungal communities (Fig. 2A,C).

The analysis of species richness within bacterial communities revealed that 9.8% (n = 183) of ASVs were consistently present in samples from all three regions (Fig. 2B). Moreover, 11.2% (n = 210) of ASVs were unique to the BGC, 26.8% (n = 500) were unique to the XGYZ, and 36.6% (n = 683) were unique to the NMC. In the case of fungal communities, 7.7% (n = 25) of ASVs were consistently found in samples from all three seasons (Fig. 2D). Additionally, 20.7% (n = 67) of ASVs were unique to the BGC, 29.7% (n = 96) were unique to the XGYZ, and 28.8% (n = 93) were unique to the NMC. These findings indicate that endophyte communities within *S. chamaejasme* roots exhibit significant regional variations in bacterial and fungal species richness.

**Figure 3:**
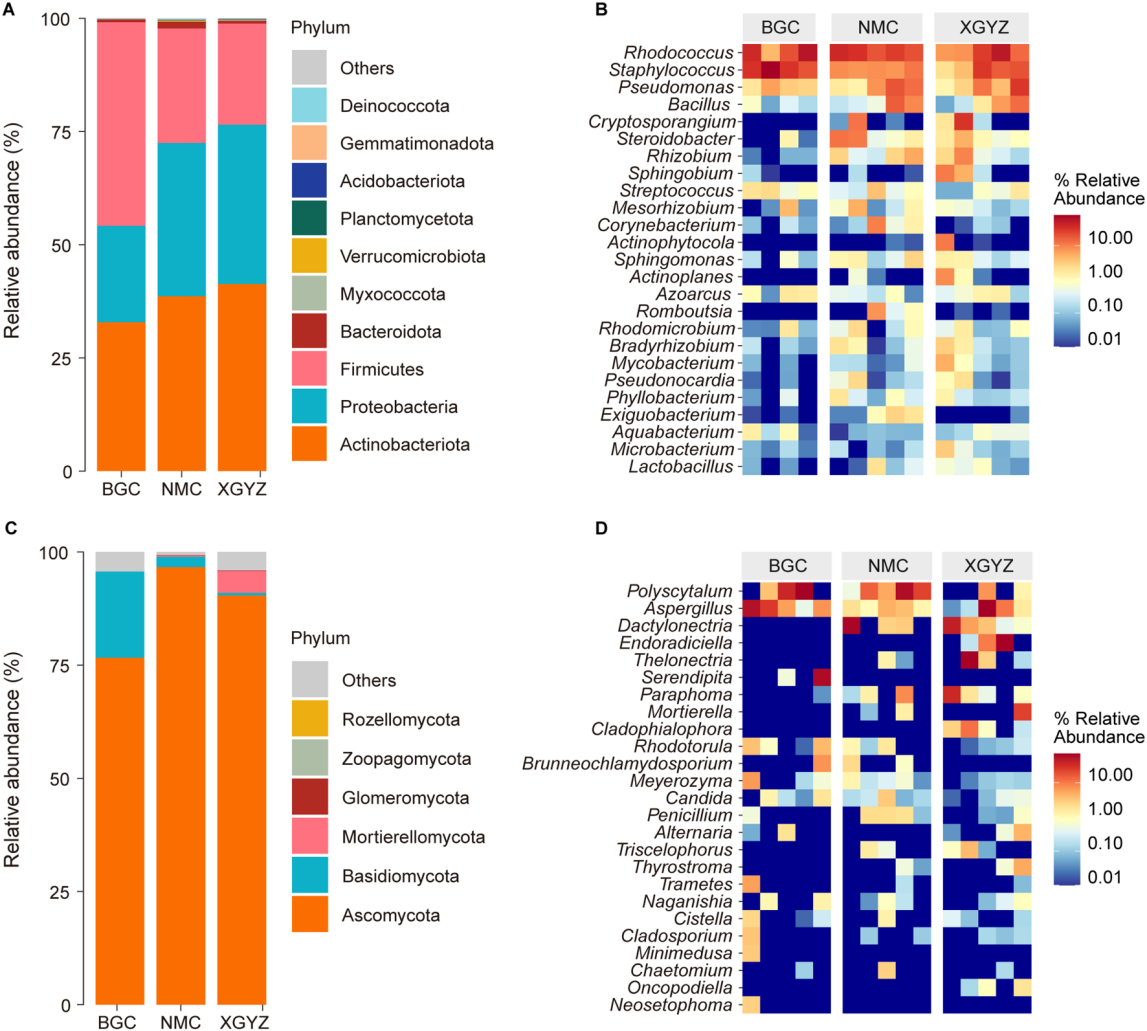
Composition of endophytes in *S. chamaejasme* root samples. (**A**) Community composition of endophytic bacteria at the phylum level. (**B**) Community composition of endophytic bacteria at the genus level. (**C**) Community composition of endophytic fungi at the phylum level. (**D**) Community composition of endophytic fungi at the genus level.

To investigate the regional dynamics of endophyte communities in *S. chamaejasme* roots, we analyzed the trends in the relative abundance of different regions. Initially, we focused on the bacterial community composition in various regions (Fig. 3A,B). At the phylum level, within the BGC, the dominant taxa (with a relative abundance exceeding 1%) and their mean relative abundances are as follows: Firmicutes (44.95%), Actinobacteria (32.86%), and Bacteroidetes (1.51%). In the NMC, the mean relative abundances of the dominant phyla were as follows: Actinobacteria (38.6%), Proteobacteria (33.87%), Firmicutes (25.3%), and Bacteroidetes (1.51%). In the XGYZ, the mean relative abundances of the dominant phyla were as follows: Actinobacteria (41.3%), Proteobacteria (35.2%), and Firmicutes (22.3%). At the genus level, within the BGC, the dominant taxa (with a relative abundance exceeding 1%) and their mean relative abundances are as follows: *Rhodococcus* (27.0%), *Staphylococcus* (20.2%), and *Pseudomonas* (35.8%). In the NMC, the mean relative abundances of the dominant genera were as follows: *Rhodococcus* (38.6%), *Staphylococcus* (33.9%), *Pseudomonas* (25.3%), *Bacillus* (5.6%) and *Cryptosporangium* (1.51%). In the XGYZ, the mean relative abundances of the dominant phyla were as follows: *Rhodococcus* (22.6%), *Staphylococcus* (10.4%), *Pseudomonas* (10.0%), *Bacillus* (4.5%) and *Steroidobacter* (1.9%).

Second, we focused on the fungal community composition in various regions (Fig. 3C,D). At the phylum level, within the BGC, the dominant taxa and their mean relative abundances were as follows: Ascomycota (76.6%), Basidiomycota (19.0%) and Glomeromycota (1.51%). In the NMC, the mean relative abundances of dominant phyla were as follows: Ascomycota (96.7%) and Basidiomycota (2.26%). In the XGYZ, the mean relative abundances of the dominant phyla were as follows: Ascomycota (90.4%) and Glomeromycota (4.9%). At the genus level, within the BGC, the dominant taxa and their mean relative abundances are as follows: *Polyscytalum* (22.2%), *Aspergillus* (19.3%), *Serendipita* (12.2%), *Rhodotorula* (1.7%), *Meyerozyma* (1.5%), *Brunneochlamydosporium* (1.4%) and *Trametes* (1.2%). In the NMC, the mean relative abundances of the dominant phyla were as follows: *Polyscytalum* (52.6%), *Dactylonectria* (14.2%), *Aspergillus* (2.5%), *Paraphoma* (1.9%) and Penicillium (1.3%). In the XGYZ, the mean relative abundances of the dominant phyla were as follows: *Aspergillus* (18.1%), *Endoradiciella* (15.8%), *Thelonectria* (14.3%), *Dactylonectria* (10.8%), *Paraphoma* (7.9%), *Mortierella* (4.7%), *Cladophialophora* (3.0%), *Polyscytalum* (1.7%), *Thyrostroma* (1.2%) and *Alternaria* (1.0%).

For the bacterial community, the dominant phylum at the phylum level was almost the same in all three samples, with the difference that Proteobacteria was not found in the dominant phylum of BGC and Bacteroidetes was not found in the dominant phylum of XGYZ. In addition to this, the dominant phylum of NMC and XGYZ is Actinomycetes, and its dominant phylum was Firmicutes in BGC. At the genus level, the dominant genus was *Rhodococcus* in all three samples. For the fungal community, the dominant phylum at the phylum level was almost the same in all three samples, and the dominant phylum was Ascomycota in all three samples, with the difference that no Glomeromycota was found in NMC, and no Bacteroidetes was found in XGYZ. At the genus level, the main dominant genus in all three samples was *Polyscytalum* in BGC and NMC, *Aspergillus* in XGYZ.

In summary, we identified the predominant endophytes in *S. chamaejasme* root samples as *Polyscytalum*, *Aspergillus* and *Serendipita* within the fungal community and *Rhodococcus*, *Staphylococcus* and *Pseudomonas* within the bacterial community.

### The differences in the endophyte communities in *S. chamaejasme* roots

**Figure 4:**
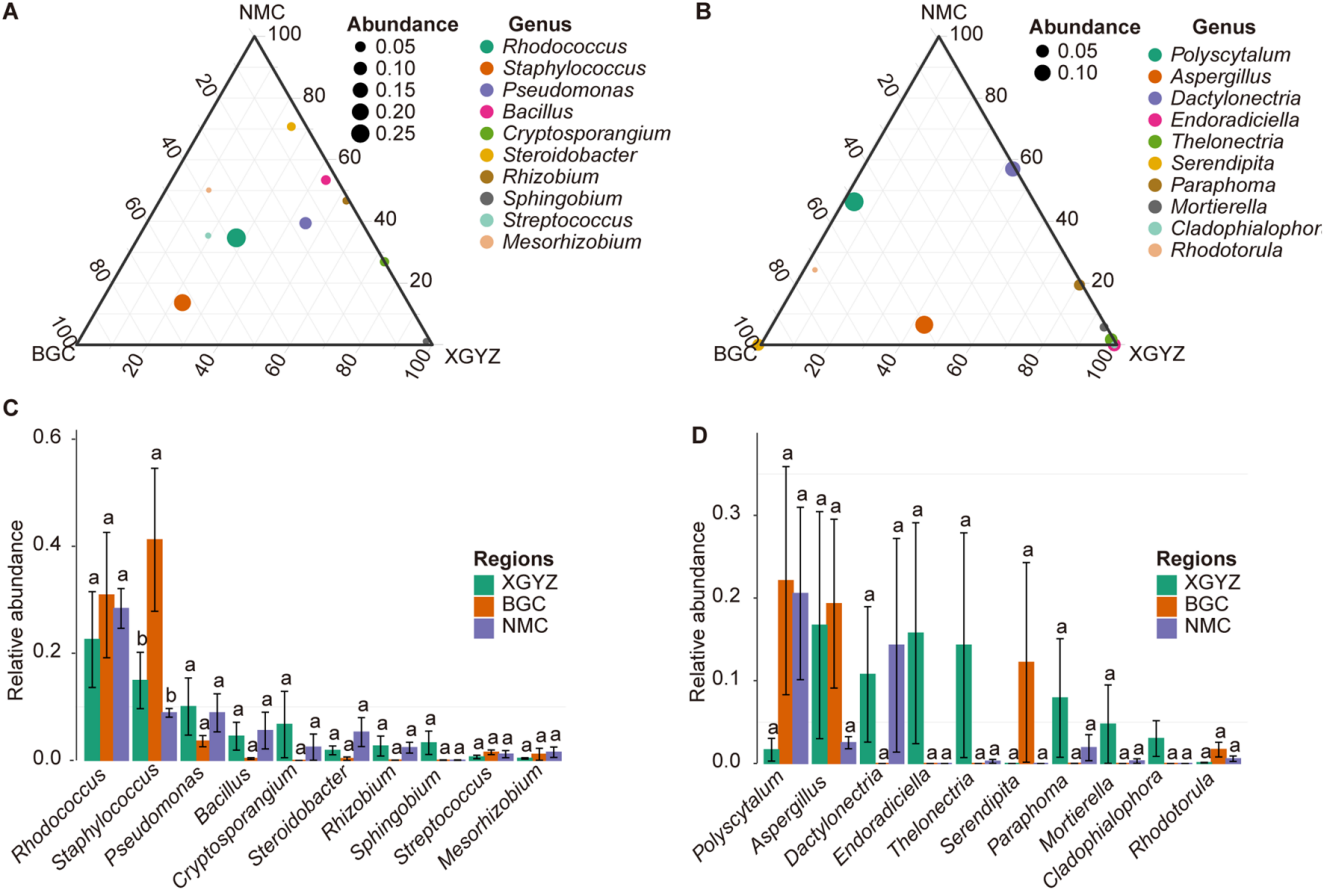
Regional Variations in endophyte Communities within *S. chamaejasme* L. roots. (**A**) Relative abundances in the bacterial community across different regions. **(B)** Relative abundances in the fungal community across different regions. **(C)** Differential analysis of relative abundances in the bacterial community among different regions. **(D)** Differential analysis of relative abundances in the fungal community among different regions.

We conducted a comprehensive analysis to investigate regional variations in endophyte communities within *S. chamaejasme* roots (Fig. 4A,B). For the bacterial communities, we observed that the relative abundance of *Rhodococcus* remained consistent across all three regions. In contrast, *Staphylococcus* exhibited a significantly higher relative abundance in BGC than in NMC and XGYZ, with a statistical significance of p < 0.05 (Fig. 4C). Additionally, *Pseudomonas*, *Bacillus*, *Cryptosporangium*, *Steroidobacter*, *Rhizobium*, *Streptococcus*, and *Rhizobium* also displayed higher relative abundances in BGC than in NMC and XGYZ. In contrast, *Sphingobium* exhibited a higher relative abundance in XGYZ relative to both NMC and BGC. The relative abundances of *Streptococcus* and *Mesorhizobium* were higher in BGC and NMC than in XGYZ.

Regarding fungal communities (Fig. 4B), we observed that the relative abundance of *Polyscytatum* displayed higher values in BGC than in NMC and XGYZ (Fig. 4D). Similarly, *Aspergillus* exhibited greater relative abundances in BGC and XGYZ than in NMC. On the other hand, *Dactylonetria* had a higher relative abundance in NMC and BGC relative to XGYZ. The relative abundances of endoradictria, *Theleonectria*, *Paraphoma*, and *Mortierella* were higher in XGYZ and NMC than in BGC. The relative abundance of *Rhodotorula* was higher in both BGC and NMC than in XGYZ, although these differences did not reach statistical significance (Fig. 4D).

### Co-occurrence network of endophyte communities in *S. chamaejasme*

**Figure 5:**
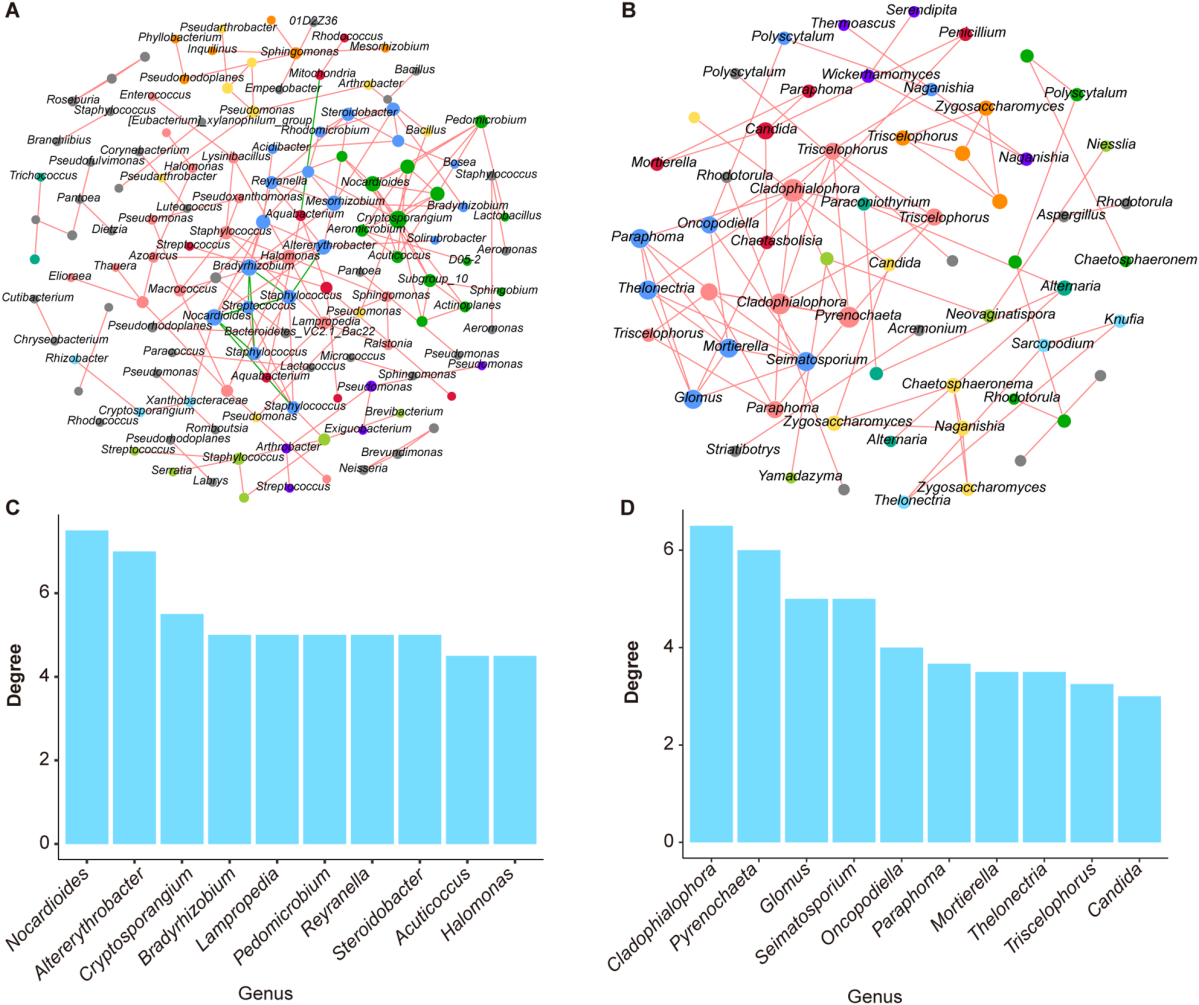
Co-occurrence network pattern. (**A**) Co-occurrence networks of endophytic bacterial communities in *S. chamaejasme* roots; modules of the network are shown in different colours. (**B**) Co-occurrence networks of endophytic fungal communities in *S. chamaejasme*; modules of the network are shown in different colours. **(C)** The value of the mean degree in the co-occurrence networks of endophytic bacterial communities in *S. chamaejasme* roots. **(D)** The value of the mean degree in the co-occurrence networks of endophytic fungal communities in *S. chamaejasme* roots.

Co-occurrence network analysis investigated potential relationships within endophyte communities in *S. chamaejasme* roots (Fig. 5A,B). The modularity coefficients for all co-occurrence networks exceeded 0.4, indicating evident modularity (Table 2). Differences in node and edge numbers were observed, suggesting dynamic variations between bacterial and fungal communities. Analysis of both bacterial and fungal communities revealed that the co-occurrence network for bacterial communities had a higher total number of edges and average degree (the average number of connections for all nodes in the network). Furthermore, bacterial communities exhibited a lower average clustering coefficient, network diameter, and modularity. In summary, the co-occurrence network of bacterial communities appeared to be more intricate, signifying a tighter interplay among them.

**Table 2:** Topological characteristics of co-occurrence networks in Bacterial and Fungal communities.

	Node number	Edge number	Average degree	Network diameter	Average path length	Modularity	Average clustering coefficient
Bacterial	135	164	2.43	0.01	3.92	0.82	0.40
Fungal	62	77	2.48	0.04	2.29	0.83	0.65

Furthermore, we identified high-degree nodes within the co-occurrence networks, as illustrated in Fig. 5C,D. In the bacterial community co-occurrence network, the top 10 genera with the highest degree were *Nocardioides*, *Altererythrobacter*, *Cryptosporangium*, *Bradyrhizobium*, *Lampropedia*, *Pedomicrobium*, *Reyranella*, *Steroidobacter*, *Aculticoccus*, and *Halomonas*. In the fungal community co-occurrence network, the top 10 genera with the highest degree were *Cladophialophora*, *Pyrenochaeta*, *Glomus*, *Seimatosporium*, *Oncopodiella*, *Paraphoma*, *Mortierella*, *Thelonectria*, *Triscelophorus*, and *Candida*. These findings suggest that these genera play crucial roles in the endophytic communities of *S. chamaejasme*.

## Discussion

This study used amplicon sequencing technology to conduct high-throughput sequencing and analysis of endophytic bacteria and fungi from three regions of Tibet (BGC, NMC, and XGYZ), highlighting key findings regarding diversity and community composition. A total of 876,297 high-quality sequences were obtained for bacterial communities, classified into 1868 ASVs. For fungal communities, 9,120,801 high-quality sequences were obtained, classified into 323 ASVs. The diversity of bacterial communities was highest in NMC, followed by XGYZ and BGC. The diversity of fungal communities was also highest in NMC. The annual precipitation in the NMC area is 410 mm, the annual precipitation in the BGC area is 289–390 mm, and the annual precipitation in the XGYZ area is 290–321 mm. The annual precipitation in the NMC area is the highest among the three sample points, indicating that precipitation and endophyte diversity are closely related. Previous studies have shown that differences in precipitation will lead to differences in soil microorganisms, and annual precipitation is positively correlated with soil microbial diversity^42,43^. In addition, among the three sample points, the NMC sample point has the highest pH and salinity value (pH 8.5, salinity 3 ms/cm), followed by XGYZ (pH 8.2, salinity 0.05 ms/cm). cm) and BGC (pH 7.4, salinity 0.11 ms/cm)^44,45^. Correspondingly, the Shannon diversity index NMC of the endophyte *S. chamaejasme* is the highest, followed by XGYZ and BGC; the ACE index NMC is the highest, followed by XGYZ and BGC; the richness index (especially bacteria) is also the highest in NMC, followed by XGYZ and BGC; this shows that pH and salinity are also closely related to the community structure and diversity of endophytic bacteria in *S. chamaejasme*. This is similar to previous research results. Under the stress of high salinity, plants require a large number of endophytes (including bacteria and fungi) to colonize to help plants grow under high-salt conditions, thus causing soil salinity. Positive correlation with endophyte diversity^46,47^. As for pH, soil pH is also the main determinant of endophytic bacterial community structure and diversity. For example, Lauber et al. pointed out that soil pH will significantly affect the community composition of endophytic bacteria. When pH exceeded 8.8, endophytic bacterial community diversity decreased with increasing pH, and bacterial diversity was significantly negatively correlated with soil pH^48^. In the endophytic fungal community, there is no significant correlation between pH and endophytic fungal diversity^49^. This also seems to explain why the diversity of endophytic bacteria in NMC was the highest in our study, while the difference in community diversity of endophytic fungi was not very obvious. Since most plant endophytes enter the plant from the soil through roots, the community composition of plant endophytes is greatly affected by the composition of soil microorganisms^50^.

The study also analyzed the endophytic bacterial and fungal community compositions of *S. chamaejasme* in different regions (BGC, NMC and XGYZ) and evaluated their differences through principal coordinate analysis (PCoA) and permutational multivariate analysis of variance (PERMANOVA). The results showed statistically significant differences in bacterial and fungal community composition across the three regions. A total of 9.8% of ASVs in the bacterial community were present in all three regions, while 11.2%, 26.8% and 36.6% of ASVs were specific to the BGC, XGYZ and NMC regions, respectively. This highlights the apparent regional specificity. In the fungal community, 7.7% of ASVs coexisted in all three regions. In comparison, 20.7%, 29.7%, and 28.8% of ASVs were unique to the BGC, XGYZ, and NMC regions, respectively, indicating that regional differences in fungi were also significant. These results indicate significant differences in the species richness of endophytic bacterial and fungal communities in different regions, and there are also region-specific ASVs, suggesting that environmental factors (such as precipitation) may affect the composition and diversity of these endophytic bacterial communities. A study by Araya et al. on the diversity and community composition of root endophytes associated with *Aristolochia chilensis* in the Atacama Desert drought gradient showed that the diversity and community composition of *A. chilensis* endophytes are related to differences in drought levels across the gradient, which is related to the results of this study being similar^51^. In addition, during the long-term co-evolution process with host plants, plant endophytes have developed their unique adaptation characteristics such as host conservation and geographical specificity^41^, which is also one of the reasons for the above results. Existing studies have shown that not only different plants have different endophytic community structures, but also there are generally differences in the endophytic community structures of the same species under different geographical conditions, such as the medicinal plants *Codonopsis pilosula*, *Astragalus membranaceus*, and *Forsythia suspensa* on the Qinghai-Tibet Plateau. Such geographical differences also exist in the diversity of endophytes^42^.

Furthermore, this study investigated the dynamics of the endophytic community of *S. chamaejasme* in different regions, paying particular attention to the composition of bacterial and fungal communities. We analyzed bacterial and fungal abundance changes in the BGC, NMC and XGYZ regions. The results revealed the dominant endophytes in *S. chamaejasme* root samples from different regions. At the phylum level, in the bacterial community, Actinobacteria, Proteobacteria and Bacteroidota were the dominant phyla. Actinobacteria is the core microbial group in the endophytic bacteria of *S. chamaejasme*, which is similar to the research results of Zhao et al.^52^ Actinobacteria is also the dominant phylum in the medicinal orchid *Dendrobium*. In this study, Actinobacteria were also identified as the core phylum of endophytic bacteria in roots, suggesting that *S. chamaejasme* may recruit actinomycetes from the environment into its roots by its secondary metabolites. Relevant studies have shown that many endophytic actinomycetes can promote plant growth^53,54^, indicating that actinomycetes may play an important role in the growth of *S. chamaejasme*. In the fungal community, Ascomycota was the dominant phylum. This is consistent with the results of Jin et al.’s determination of endophytic fungi in the roots of *S. chamaejasme* in Cuiying Mountain, Yuzhong County, Lanzhou, and the dominant phylum is Ascomycota^36^. At the genus level, in the bacterial community, *Rhodococcus*, *Staphylococcus*, *Pseudomonas*, and *Bacillus* were the dominant genera, while in the fungal community, *Polycystis* and *Aspergillus* were the dominant genera. Abundant taxa may show more robust environmental adaptability. These findings also highlight differences in endophyte community composition between regions, with specific genera being more abundant in specific regions. *Pseudomonas* belongs to the phylum Proteobacteria, and previous studies have shown that *Pseudomonas* can produce siderophores^55^, dissolve phosphate^56^, fix nitrogen^57^, and transport soil nutrients into plants to promote plant growth. grow and protect plants against pathogenic bacteria^58^, suggesting that *Pseudomonas* species, which are dominant in *S. chamaejasme*, may play an important role in *S. chamaejasme* growth and disease resistance, thereby enhancing its ability to compete for habitat. Tang et al.^59^ isolated *Aspergillus* by studying the endophytic fungus of *S. chamaejasme* and found that it could significantly increase the biomass of *S. chamaejasme*. These dominant microbiota found in the roots of *S. chamaejasme* may promote its growth and stress resistance, which can provide reference for the subsequent development of related biofertilizers and the prevention and control of *S. chamaejasme*. As for the relationship between other endophytes and *S. chamaejasme*, further research is needed.

This study focused on the differences in root endophyte communities, including bacteria and fungi, of *S. chamaejasme* in different regions. The research uncovered some interesting trends. Regarding bacterial communities, the relative abundance of *Rhodococcus* was similar in all regions, indicating that it is a core member. However, the relative abundance of *Staphylococcus* was significantly higher in the BGC region, suggesting regional specificity. In addition, several other bacterial genera were also relatively more abundant in the BGC region, indicating that they are more adapted to the environmental conditions in this region. The relative abundance of the *Sphingomyces* genus was higher in the XGYZ region, indicating its adaptability. The relative abundance of *Streptococcus* and *Rhizobium* was relatively high in the BGC and NMC regions, indicating that they are commonly distributed in these two regions. In terms of fungal communities, *Polyscytatum* had a higher relative abundance in the BGC region, indicating its regional preference. *Aspergillus* had a higher relative abundance in BGC and XGYZ, indicating that it was easier to grow. The relative abundance of *Dinophylla* was higher in NMC and BGC, indicating that it is more common in these two areas. *Endoradictria*, *Theleonectria*, *Paraphoma* and *Mortierella* had higher relative abundances in XGYZ and NMC, indicating that they are more common in these regions. *Rhodotorula* was relatively more abundant in BGC and NMC, although the difference was insignificant. These results highlight the regional preferences and adaptations of some bacterial and fungal genera. Differences between regions may be influenced by the environment, soil conditions and plant interactions, and a deeper understanding of these differences can help provide a more complete understanding of plant‒microbe relationships and their potential impact on plant health and ecosystem function.

This study also used co-occurrence network analysis to study the potential relationships between endophytic communities in the roots of *S. chamaejasme*. The modularity coefficients of all co-occurrence networks exceeded 0.4, indicating the existence of apparent modularity and reflecting differences in ecological niches or functions. Differences in the number of nodes and edges between bacterial and fungal communities may be affected by time or environmental factors. Bacterial co-occurrence networks have more edges and interactions than fungi. Bacterial communities exhibited a lower average clustering coefficient, network diameter, and modularity. A lower clustering coefficient indicates a more dispersed network structure, while a smaller network diameter means shorter paths for information or resource exchange. Lower modularity indicates that the bacterial community is tightly coupled with more cooperative or shared functions. The top 10 bacterial genera with the highest number of nodes in the co-occurrence network were *Nocardia*, *Alternobacterium*, *Cryptosporidium*, *Bradyrhizobium*, *Lamprichia*, *Pedomicrobia*, *Leyla*, *Steroidobacter*, *Coccus* and *Halomonas*. *Nocardia* in *Ginkgo biloba*^60^, *Dracaena*^61^, *Coffea arabica*^62^ and other plants have been found to have antibacterial properties^63^, cytotoxic activity^61^, nematicidal activity^62^ and other activities. In the fungal community co-occurrence network, the top 10 genera with the highest number of nodes were *Cladophialophora*, *Pyrenochaeta*, *Glomus*, *Seimatosporium*, *Oncopodiella*, *Parapoma*, *Mortierella*, *Thelonectria*, *Triselophorus* and *Candida*. Among them, *Cladophialophora* is the dominant genus of endophytic fungi in the roots of red and green amaranth^64^. *Cladophialophora chaetospira* can alleviate strawberry wilt disease and promote strawberry growth^65^. These findings indirectly reveal the importance of endophytic bacteria and fungi to the growth and adaptation of *S. chamaejasme*, which is critical to understanding their complex relationships and ecosystem impacts.

## Conclusion

This study used high-throughput sequencing technology to analyze the community composition and diversity of root endophytes of *S. chamaejasme* in different locations in Tibet, providing a reference for the in-depth exploration of Rhizophora endophyte resources and the control of noxious weeds in grasslands on the Qinghai-Tibet Plateau. The study found that bacterial and fungal diversity indicators (such as the Shannon index, Pilou index, richness index and ACE index) at the NMC site were higher than those at the XGYZ and BGC sites, indicating that the endophyte diversity at the NMC site was higher. The study also found that Ascomycota was the main phylum in endophytic fungal communities in different locations, while Actinobacteria, Proteobacteria and Firmicutes were the main phyla in endophytic bacteria. At the genus level, *Polyscytalum*, *Aspergillus*, *Serendipita*, etc., dominate endophytic fungi, while *Rhodococcus*, *Staphylococcus*, *Pseudomonas*, etc., are dominant genera among endophytic bacteria. Principal coordinate analysis and PERMANOVA showed significant differences in the composition of bacterial and fungal communities in different locations. Co-occurrence network analysis of endophytes showed a positive correlation between fungal species. At the same time, there were some negative correlations among bacteria, and the cooccurrence network of bacteria was relatively more complex. These research results support the future isolation and identification of beneficial microorganisms in the roots of Daphne *S. chamaejasme* and provide a reference for grassland degradation and grassland ecosystem restoration caused by the invasion of Daphne *S. chamaejasme*. The relationship between the core flora and the active ingredient *S. chamaejasme* and the relationship between the core flora and the invasion of Daphne lupus (making it the dominant plant) deserve further study.

### Supplementary Information

Supplementary Information.

## Data Availability

The data presented in the study are deposited in the National Genomics Data Center repository, accession number CRA013215 (endophytic bacteria) and CRA013209 (endophyte).

## Abbreviations

*S. chamaejasme*: *Stellera chamaejasme* L.
ASVs: Amplicon sequence variants
PCoA: Principal coordinate analysis
NMDS: Nonmetric multidimensional scaling
