# Supplementary material for: High-throughput sequencing-based analysis of the composition and diversity of the endophyte community in roots of Stellera chamaejasme
Source: Sci Rep. 2024 Apr 13;14:8607. doi: 10.1038/s41598-024-59055-x (PMC11016073; doi:10.1038/s41598-024-59055-x)
Supplement: Supplementary file 1 — Supplementary Information. [file 41598_2024_59055_MOESM1_ESM.docx]

**Figure S1 ASV rarefaction curves(The picture on the left shows bacteria and the picture on the right shows fungi.)**


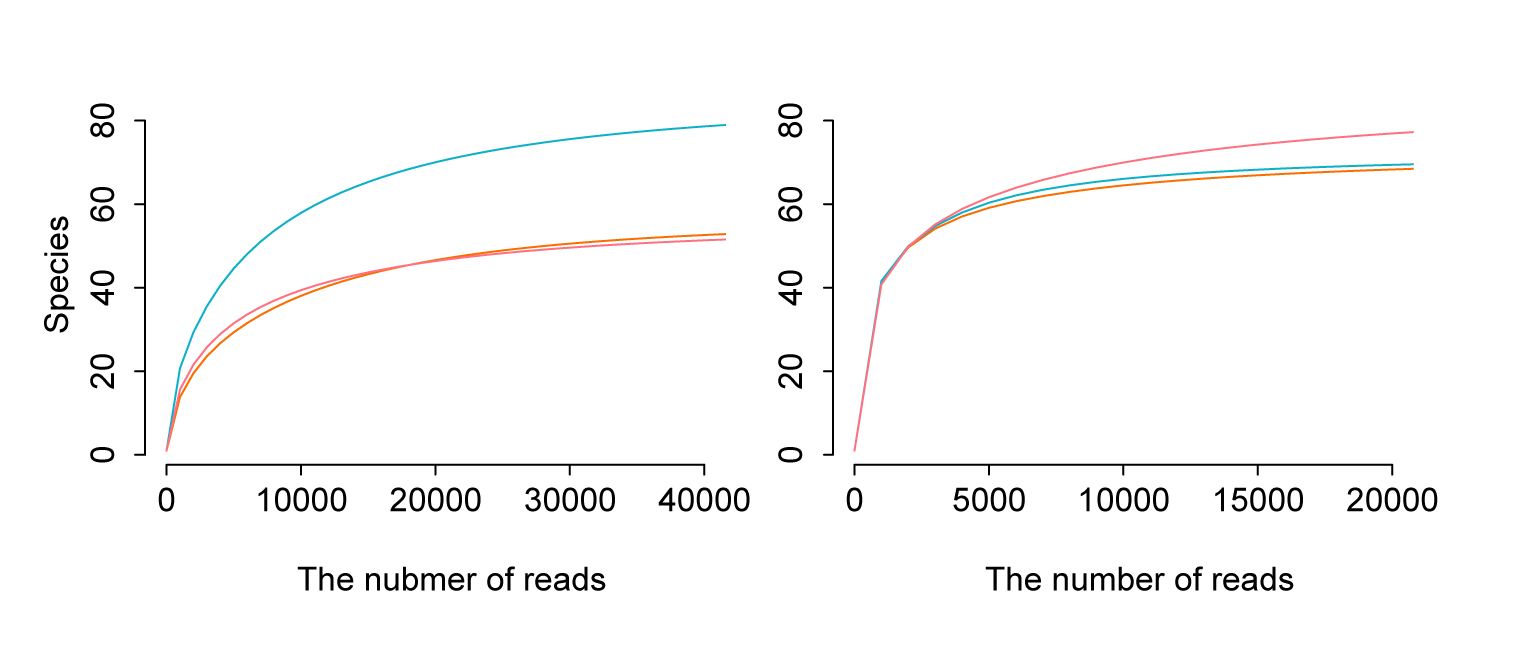


**Table S1 α diversity of endophytic bacteria in *S.chamaejasme* roots**

|  | Richness | Shannon | Simpson | Pielou | Chao1 | ACE | goods_coverage |
| --- | --- | --- | --- | --- | --- | --- | --- |
| LD_1_1 | 384 | 4.4144 | 0.9606 | 0.7418 | 384.0000 | 384.0000 | 1.0000 |
| LD_1_2 | 328 | 3.5774 | 0.8876 | 0.6175 | 328.0000 | 328.1608 | 1.0000 |
| LD_1_3 | 298 | 3.3146 | 0.8853 | 0.5818 | 301.0000 | 299.1397 | 0.9998 |
| LD_1_4 | 195 | 2.2687 | 0.6827 | 0.4303 | 196.0000 | 195.7923 | 0.9999 |
| LD_1_5 | 317 | 3.4357 | 0.9227 | 0.5966 | 320.5000 | 318.7129 | 0.9998 |
| LD_2_1 | 200 | 2.5093 | 0.7915 | 0.4736 | 200.7500 | 200.5602 | 0.9999 |
| LD_2_2 | 101 | 1.7390 | 0.5877 | 0.3768 | 102.5000 | 102.3374 | 0.9999 |
| LD_2_3 | 288 | 3.3702 | 0.9031 | 0.5951 | 288.0000 | 288.0000 | 1.0000 |
| LD_2_5 | 204 | 2.1139 | 0.6429 | 0.3975 | 204.1667 | 204.3665 | 0.9999 |
| LD_3_1 | 331 | 3.2199 | 0.8297 | 0.5549 | 332.5000 | 332.2524 | 0.9998 |
| LD_3_2 | 320 | 3.2528 | 0.8585 | 0.5639 | 321.6667 | 321.3876 | 0.9998 |
| LD_3_3 | 413 | 4.3420 | 0.9468 | 0.7209 | 413.5000 | 413.5201 | 0.9999 |
| LD_3_4 | 297 | 3.3364 | 0.8881 | 0.5860 | 299.8000 | 299.0118 | 0.9998 |
| LD_3_5 | 406 | 4.1336 | 0.9400 | 0.6882 | 410.2000 | 407.4022 | 0.9998 |

**Table S2 α diversity of endophytic fungi in *S.chamaejasme* roots**

|  | Richness | Shannon | Simpson | Pielou | Chao1 | ACE | goods_coverage |
| --- | --- | --- | --- | --- | --- | --- | --- |
| LD_1_1 | 30 | 1.4312 | 0.6835 | 0.4208 | 34.2 | 38.2500 | 0.9993 |
| LD_1_2 | 40 | 1.7625 | 0.7385 | 0.4778 | 43.0 | 44.9387 | 0.9994 |
| LD_1_3 | 54 | 1.2855 | 0.4819 | 0.3223 | 61.2 | 58.7354 | 0.9991 |
| LD_1_4 | 38 | 1.3488 | 0.5121 | 0.3708 | 38.0 | 38.1796 | 0.9999 |
| LD_1_5 | 69 | 2.4615 | 0.8321 | 0.5814 | 69.0 | 69.0000 | 1.0000 |
| LD_2_1 | 25 | 2.0312 | 0.7047 | 0.6310 | 25.0 | 25.0000 | 1.0000 |
| LD_2_2 | 36 | 1.6183 | 0.6480 | 0.4516 | 36.0 | 36.0000 | 1.0000 |
| LD_2_3 | 24 | 1.3077 | 0.6228 | 0.4115 | 24.0 | 24.0000 | 1.0000 |
| LD_2_4 | 33 | 1.1191 | 0.4890 | 0.3201 | 33.6 | 34.1035 | 0.9997 |
| LD_2_5 | 38 | 2.1108 | 0.7472 | 0.5803 | 38.0 | 38.0000 | 1.0000 |
| LD_3_1 | 41 | 1.4764 | 0.5467 | 0.3976 | 42.5 | 42.5052 | 0.9997 |
| LD_3_2 | 51 | 2.3566 | 0.8464 | 0.5994 | 51.0 | 51.3541 | 0.9999 |
| LD_3_3 | 43 | 2.4172 | 0.7725 | 0.6427 | 43.0 | 43.0000 | 1.0000 |
| LD_3_4 | 37 | 1.7231 | 0.6295 | 0.4772 | 37.0 | 37.0000 | 1.0000 |
| LD_3_5 | 33 | 1.6538 | 0.7145 | 0.4730 | 33.0 | 33.0000 | 1.0000 |
